# THE TYPES AND CONSISTENCY OF PARTICIPANT-SPECIFIED PROXIES DURING THE SPRINT CLINICAL TRIAL

**DOI:** 10.1093/geroni/igad104.2959

**Published:** 2023-12-21

**Authors:** Kat Coschigano, Sarah Gaussoin, Byron Jaeger, Stephen Rapp, Jeff Williamson

**Affiliations:** Somers High School, Katonah, New York, United States; Wake Forest School of Medicine, Winston Salem, North Carolina, United States; Wake Forest School of Medicine, Winston Salem, North Carolina, United States; Wake Forest School of Medicine, Winston Salem, North Carolina, United States; Wake Forest School of Medicine, Winston Salem, North Carolina, United States

## Abstract

**Background:**

For clinical trials or patient care, reports from a person familiar with the trajectory of a participant’s cognition are needed to ascertain incident Mild Cognitive Impairment and dementia. Informants offer a more unbiased evaluation of cognitive status. Recent large clinical trials studies, such as SPRINT, provide information about the contributions and consistency of informants throughout extended follow-up.

**Methods:**

SPRINT participants named proxies and relationship at study onset. Proxies provided information for the Functional Activities Questionnaire (FAQ) and Dementia Questionnaire (DQ). We collapsed proxy relationships into seven categories and evaluated the proportion of proxies in each category at baseline, how frequently the same proxy was retained to the last visit, and the frequency of successful proxy contact during the trial.

**Results:**

Of the 9,361 randomized SPRINT participants, 7,586 participants listed a proxy relationship from which we identified 7 categories. Participants were 35.6% female, with baseline mean age of 67.9 years. The proportion of proxies in each category was spouse/partner (51.7%), child (23.6%), sibling (8.7%), other family member (6.2%), and unelated (8.8%). The overall consistency of proxy continuation between the baseline and final SPRINT visit (mean 5.1 years) was 90.11%. Proxy consistency from first visit to last visit for a child was 93.41%, spouse/partner (91.87%), other family member (80.1%), brother/sister (86.2%), non-related (84.65%), and parent (81.73%).

**Conclusion:**

Immediate family members (spouse, child, or sibling) comprised the majority of proxies in SPRINT. Importantly, more than 90% of identified proxies at baseline continued to be available over the life of a 5-year trial.

